# The Impact of Math-Gender Stereotypes on Students’ Academic Performance: Evidence from China

**DOI:** 10.3390/jintelligence12080075

**Published:** 2024-08-01

**Authors:** Yilei Luo, Xinqi Chen

**Affiliations:** School of Economics, Renmin University of China, Beijing 100872, China; luoyilei99@sina.com

**Keywords:** math-gender stereotypes, academic performance, gender, STEM

## Abstract

This study investigates the impact of math-gender stereotypes on students’ academic performance using data from the China Education Panel Survey (CEPS), which surveyed nationally representative middle schools in China. Our sample comprises over 2000 seventh-grade students, with an average age of 13 and a standard deviation of 0.711. Among these students, 52.4% are male, and 47.6% are female. Employing a fixed effects model and instrumental variable, our findings are as follows. First, over half of the male students believe that boys are better at math than girls, and they also perceive that their parents and society hold the same belief. In contrast, fewer than half of the female students hold this belief or perception. Intriguingly, among these students, female math performance surpasses that of males. Second, stereotypes hinder female math performance, especially among low-achieving ones, while benefiting high-achieving male students. Finally, perceptions of societal stereotypes have the greatest effect on math performance, followed by self-stereotypes and perceptions of parental stereotypes. Understanding the implications of these findings highlights the importance of addressing math-gender stereotypes to promote equal participation and success for both genders in STEM fields.

## 1. Introduction

A gender stereotype is a widely held belief or assumption about the traits, behaviors, and roles society deems appropriate for men and women (Cusack 2013). These societal expectations directly influence the cognition and behavior of both genders, subtly shaping individual development. In educational settings, gender stereotypes often manifest as the belief that “boys are better at math than girls” (Master 2021). This stereotype significantly impacts girls’ behavior and, subsequently, their math performance (Spencer et al. 1999). 

Math performance is a reliable indicator of readiness for STEM disciplines and can predict future job market success (Master 2021; Altonji and Blank 1999; Card and Payne 2021; Joensen 2009). Females’ disadvantage in math may lead to discrimination in the job market. LinkedIn data highlights the underrepresentation of women in fields such as engineering, manufacturing, construction, information technology, and telecommunications. Experimental studies have also confirmed the existence of employment discrimination (Reuben et al. 2014). The lack of gender diversity in these industries not only hinders women’s career development but also stifles innovation and returns (Paglin and Rufolo 1990). Therefore, investigating whether gender stereotypes influence students’ math performance is crucial. On one hand, it helps address whether the gender differences in math performance are due to inherent ability differences or are shaped by postnatal environments. On the other hand, it provides insights into gender discrimination in the job market.

Existing literature has explored the impact of math-gender stereotypes on math performance from various dimensions.

Firstly, concerning the independent variables, studies have examined the effects of math-gender stereotypes from different sources, including self-perception, teachers, parents, peers, and society. For example, Bagès and Martinot (2011) found that self-endorsed math-gender stereotypes lower girls’ math performance (Bagès and Martinot 2011), while Dossi et al. (2021) found that mothers’ attitudes toward female social roles are correlated with girls’ math test scores (Dossi et al. 2021). However, no study has yet used the same sample to conduct a unified analysis and comparison of stereotypes from different sources. Comparing the impact of these sources is crucial as it helps policymakers identify key areas to address in reducing stereotypes.

Secondly, regarding the subjects of the studies, most research focuses on the overall effects of math-gender stereotypes on boys and girls, with fewer studies further subdividing the population into groups such as high-achieving and low-achieving students (Bagès and Martinot 2011; Alan et al. 2018). Although some research has examined the impact of stereotypes on high-achieving students, the findings are not yet consistent (Good et al. 2012; Flore et al. 2018). Given the potential physiological and psychological differences within these refined groups, a more detailed analysis is necessary.

Finally, regarding the research results, findings vary significantly across different cultural backgrounds and samples. For instance, research in some countries has found that stereotype threat negatively affects girls, including studies from France (Bagès and Martinot 2011), Germany (Keller 2007; Keller and Dauenheimer 2003), Italy (Muzzatti and Agnoli 2007), Spain (Delgado and Prieto 2008), and Uganda (Picho and Stephens 2012). However, studies from other countries like the United States (Ganley et al. 2013), the Netherlands (Flore et al. 2018), and another sample in Italy have found no significant effects of stereotype threat (Agnoli et al. 2021). Additionally, most existing studies focus on Western contexts. Since math-gender stereotypes are influenced by specific social and cultural factors, independent and more detailed empirical analyses are needed for Eastern contexts.

This study leverages data from the China Education Panel Survey (CEPS) to investigate the impact of math-gender stereotypes on students’ math performance within the Chinese context. CEPS employed stratified random sampling to select a representative sample from over 100 schools in China, surveying students, parents, teachers, and school administrators. The survey was conducted in two waves: the baseline survey in 2013–2014 and the follow-up survey in 2014–2015. In the baseline survey, students’ perceptions of math-gender stereotypes were collected across three dimensions, including questions such as “Do you/your parents/most people around you think that boys are better at math than girls?”.

Our final sample comprises over 2000 seventh-grade students, with an average age of 13. To study the impact of math-gender stereotypes on students’ math performance, we employed regression methods, with math-gender stereotypes as the independent variable and students’ math performance as the dependent variable. Since stereotypes are endogenous, there may be factors that simultaneously influence both stereotypes and math performance. For instance, a boy with strong math skills might also hold gender stereotypes and perform well in math. To mitigate this endogeneity issue, we leveraged the advantages of panel data and first applied a fixed effects model to reduce the impact of time-invariant variables. Additionally, we introduced a new instrumental variable: the difference in average math scores between male and female students within the same class during the first wave. This instrumental variable helped reduce endogeneity caused by time-varying factors. Through these methods, we can assert that our results provide a causal inference on the impact of stereotypes on math performance.

Given that stereotypes may affect boys and girls differently, we analyzed the impact of stereotypes on math performance separately by gender. For each gender, we further divided the sample into the full sample, the first half sample, and the second half sample. In each sample, we examined the influence of three types of stereotypes. The results of this study contribute to understanding whether stereotypes affect males and females differently, potentially providing explanations for gender gaps in the labor market and the underrepresentation of women in STEM fields.

## 2. Literature Review

### 2.1. The Gender Gap in Math Performance

Regarding the potential gender differences in math scores, global research outcomes are highly diverse (Lohman 2009; Machin and Pekkarinen 2008; Pope and Sydnor 2010). Notably, female students in the United States have achieved parity with their male counterparts in math scores, a trend observed in several other countries as well (Hyde and Mertz 2009; Hedges and Nowell 1995). However, cross-national studies indicate that in countries where societal and cultural gender stereotypes are less pronounced, the gender gap in math scores tends to be narrower (Guiso et al. 2008; Nosek et al. 2009; Else-Quest et al. 2010).

The underlying causes of the gender gap in math achievement have long been debated. Whether these disparities stem from inherent intellectual and physiological differences between males and females (Benbow and Stanley 1980) or are shaped by postnatal sociocultural influences (Nollenberger et al. 2016) remains a contentious issue. In the specific cultural context of China, it is essential to explore whether math-gender stereotypes exist in this environment and whether these stereotypes influence students’ math performance.

### 2.2. Impact of Stereotypes on Their Targets

Abundant empirical research, both domestically and internationally, has revealed that negative stereotypes have adverse effects on their targets, a phenomenon known as stereotype threat. Scholars have found that stereotype threat affects individuals’ performance through mechanisms that include inducing anxiety (Spencer et al. 1999), self-doubt (Steele and Aronson 1995), and reduced working memory capacity (Schmader and Johns 2003; Schmader et al. 2008). Under the influence of stereotypes, several mechanisms unfold: (a) stress-induced physiological responses stemming from stereotypes directly impair the functioning of the brain’s prefrontal cortex; (b) targets of stereotypes become increasingly fixated on their performance; and (c) the adverse thoughts and emotions caused by stereotypes necessitate self-regulation. This triad of mechanisms converges, imposing an additional cognitive burden on individuals, thereby depleting their finite cognitive resources and ultimately impairing their performance in other tasks (Bagès and Martinot 2011). This intricate interplay between stereotype threat and cognitive resources underscores the complexity of the impact of stereotypes on performance outcomes.

Referring specifically to the math-gender stereotype that boys are better at math than girls, these stereotypes have the potential to exert influence on girls through the aforementioned three pathways. Individual math-gender stereotypes are multidimensional, encompassing self-endorsed stereotypes as well as those perceived from various sources. These sources include teachers, parents, peers, and other individuals in their surroundings. The impacts of math-gender stereotypes from different sources on males and females may vary (Eble and Hu 2020).

### 2.3. Impact of Self-Endorsed and External Math Gender Stereotypes

#### 2.3.1. Self Math-Gender Stereotypes

Research across diverse cultural contexts has explored the impact of math-gender stereotypes on students’ math performance, yielding varied results. Many studies have focused on the concept of stereotype threat and its effects on female students’ performance. In some countries, stereotype threat has been found to negatively affect girls, including in France (Bagès and Martinot 2011), Germany (Keller 2007; Keller and Dauenheimer 2003), Italy (Muzzatti and Agnoli 2007), Spain (Delgado and Prieto 2008), and Uganda (Picho and Stephens 2012). However, other studies have found no significant effects of stereotype threat in countries such as the United States (Ganley et al. 2013), the Netherlands (Flore et al. 2018), and Italy (Agnoli et al. 2021). 

Even within the same country, different samples can yield conflicting conclusions. For instance, in Italy, Muzzatti and Agnoli (2007) found that stereotype threat impacts math performance, particularly in specific age groups, such as 13-year-old girls (average age 13, grade 8th), but not younger girls (ages 7–10, grades 2–5) (Muzzatti and Agnoli 2007). In contrast, Agnoli et al. (2021) found no significant effects of stereotype threat in their study (Agnoli et al. 2021).

In addition to exploring the impact of stereotypes across different cultural backgrounds, the literature also investigates whether stereotypes affect specific groups, such as high-achieving girls. Some studies have identified negative impacts of stereotypes on high-achieving girls (Spencer et al. 1999; Good et al. 2012), while others have found no significant effects (Flore et al. 2018). Flore et al. (2018) suggested that the absence of significant effects in some studies might be due to publication bias. For boys, math-gender stereotypes are found to have a positive effect (Alan et al. 2018; Kiefer and Sekaquaptewa 2007).

#### 2.3.2. Parental Math-Gender Stereotypes

Parental gender stereotypes about mathematics influence children’s academic performance through various channels. Firstly, these stereotypes affect parents’ expectations for their children, thereby shaping the children’s attitudes towards mathematics (Jacobs and Eccles 1991). Secondly, gender biases impact how much parents invest in their children’s education (Dossi et al. 2021). Regarding which parent has a greater influence, Tomasetto et al. (2011) found that it is mothers’ beliefs about math-gender stereotypes, rather than fathers’, that moderate the impact of math-gender stereotypes on their daughters (Tomasetto et al. 2011).

Additionally, the literature reveals that parental influence on children’s math performance is not solely driven by math-gender stereotypes but also by broader gender norms. Dossi et al. (2021) discovered that mothers’ attitudes toward female social roles correlate with girls’ math test scores but not with boys’ (Dossi et al. 2021), while Rodríguez-Planas and Nollenberger (2018) demonstrated that social gender norms affect parental expectations regarding girls’ academic knowledge relative to boys’, but not other success attributes such as non-cognitive skills (Delgado and Prieto 2008).

The impact of parental influence on children’s math performance varies across different cultural backgrounds. Gender role norms appear to explain lower math performance among girls only in relatively affluent white families, whereas they seem less relevant for the performance of black girls (Dossi et al. 2021). In China, Tang and Zhao (2024) found that girls from gender-equal regions are more likely to perceive higher educational expectations and confidence from their parents, thereby influencing their mathematics performance (Tang and Zhao 2024).

#### 2.3.3. Teacher Math-Gender Stereotypes

Social science research reveals that teachers often harbor math-gender stereotypes, perceiving math as more challenging for female students (Terrier 2020). These stereotypes can influence students’ performance through various channels: by directly affecting students’ own stereotypes (Keller 2001), impacting students’ confidence (Keller 2001), influencing teachers’ expectations of students (Rosenthal and Jacobson 1968; Lazarides and Watt 2015), and through classroom teaching and interaction (Lazarides and Watt 2015; Riegle-Crumb and Humphries 2012). 

There is substantial literature examining the effects on boys and girls separately. Among boys, the impact of teachers’ stereotypes on performance is inconclusive. Lazarides and Watt (2015) found that boys have stronger self-perceived teacher expectations, leading to higher self-expectations and more ambitious plans for math-related careers (Lazarides and Watt 2015). Riegle-Crumb and Humphries (2012) found that math teachers perceive white boys as more capable and interact with them more, thereby improving their performance (Riegle-Crumb and Humphries 2012). However, Terrier (2020) found that teachers’ math biases favor girls, leaving boys behind (Terrier 2020). Among girls, most studies found that teachers’ stereotypes reduce their performance (Carlana 2019; Rosenthal and Jacobson 1968). Specifically, Carlana (2019) found that teachers’ stereotypes exacerbate gender gaps in math performance; this widening is attributed to lowered math scores among stereotyped female students due to reduced self-confidence rather than elevated male performance (Carlana 2019).

#### 2.3.4. Societal Math-Gender Stereotypes

Adolescents’ perceptions of their mathematical abilities are influenced not only by their personal math gender stereotypes but also by their perceptions of others’ gender stereotypes. Many studies have measured whether adolescents believe others hold gender stereotypes about mathematics. Some studies have found that both male and female adolescents generally perceive others as endorsing traditional gender stereotypes favoring male over female students (Bonnot and Croizet 2007; Steffens et al. 2010; Justicia-Galiano et al. 2023). However, there are also studies that refute this viewpoint (Kurtz-Costes et al. 2014; Martinot et al. 2012).

Kurtz-Costes et al. (2014) found that children in fourth and sixth grades are more likely to favor their own gender group rather than traditional stereotypes compared to eighth graders (Kurtz-Costes et al. 2014). Martinot et al. (2012) found that French girls and boys do not perceive others as endorsing the superiority of a gender group in mathematics (Martinot et al. 2012).

Justicia-Galiano et al. (2023) indicate that when girls become aware of math gender stereotypes around them, it can negatively affect them by causing feelings of insecurity and anxiety (Justicia-Galiano et al. 2023). This phenomenon also extends to racial stereotypes about mathematics. Students who are aware of racial stereotypes they do not endorse but lack effective refutation may have poorer academic performance (Nasir et al. 2017).

## 3. Method

### 3.1. Background

The data used in this study come from the China Education Panel Survey (CEPS), meticulously designed and conducted by the National Survey Research Center (NSRC) of Renmin University of China. CEPS provides comprehensive information at various levels, including students, parents, classes, and schools, offering a rich and valuable basis for analysis in this study.

To date, the CEPS has conducted two waves of data collection: a baseline survey during the 2013–2014 academic year and follow-up data collected in 2014–2015. The baseline survey targeted two groups of students concurrently: those in the first year (7th grade) and those in the third year (9th grade) of junior high school. The follow-up data in 2014–2015 included all 10,279 students from the initial baseline survey in the first year (7th grade). More than 90% of the surveyed schools are public schools. Considering that the majority of primary and secondary schools in China are public, the sample is representative. Additionally, with approximately 50% of the sample coming from urban areas and 50% from rural areas, the sample distribution is also representative.

In the sample selection process, we first restricted the sample to 7th-grade students with tracking data available from the baseline survey, allowing the use of panel data analysis methods. Furthermore, at the school level, sample screening was conducted to mitigate endogeneity. According to CEPS sampling rules, “if the surveyed grade in a sampled school has only 1 or 2 classes, then all classes are sampled; if there are 3 or more classes, then 2 classes are randomly selected.” Based on these rules, we took the following steps: we retained schools with exactly two sampled classes and only kept those with equal average math scores across the two classes. Since some class-level attributes may also influence students’ math scores, such as the math learning atmosphere, retaining schools with classes that have equal average scores approximates the balance of class-level variables affecting math scores between the two classes, thus reducing endogeneity.

In the final sample, 52.4% of the students are male, and 47.6% are female. The average age of the students is 13 years, with a standard deviation of 0.711. At this age, the impact of stereotypes on academic performance has been explored in several countries. We aim to provide evidence from China.

### 3.2. Variable Setting and Summary Statistics

To investigate the impact of math-gender stereotypes on students’ academic performance, we designated academic scores as the dependent variable and math-gender stereotypes as the independent variables. Table 1 presents the descriptive statistics of these variables.

#### 3.2.1. Dependent Variable: Students’ Academic Performance

In addition to survey variables, CEPS collected students’ midterm exam scores in math, Chinese, and English for the fall semesters of 2013–2014 (baseline survey) and 2014–2015 (follow-up survey) from the surveyed schools. We standardized the scores for each subject at the school level. Utilizing data from two waves, we employed a fixed-effects model for analysis. The standardized student math scores were used as the dependent variable in the primary model, while the standardized Chinese scores were used in the placebo test. 

For descriptive analysis, examining the raw scores provides a clearer picture of the actual performance of male and female students. Therefore, Table 1 displays the raw scores. In the first wave of the entire sample, the average math score was 75.274 (out of 150), with female students outperforming male students: the average math score for females was 77.446, while for males, it was 73.293. In the second wave of the entire sample, the average math score was 75.188, and again, female students outperformed male students: the average math score for females was 73.540, while for males, it was 71.423. Therefore, our results show that female students outperformed male students in math. It is important to note that our sample consists of seventh-grade students with an average age of 13. Many studies have found that gender differences in math become more pronounced in high school (Muller 1998; Leahey and Guo 2001), with males’ advantage in calculations becoming apparent between the ages of 15 and 27 (Borgonovi et al. 2021). In China, evidence also indicates that the gap between male and female students widens in high school (Zhu et al. 2018). Therefore, this sample does not represent gender differences in math performance across all age groups in China.

#### 3.2.2. Independent Variable: Math-Gender Stereotype

The China Education Panel Survey (CEPS) included questions about math-gender stereotypes only in the baseline survey, not in the follow-up survey. The baseline survey asked three questions: “Do you/your parents/most people around you believe that boys are better at learning math than girls?” These questions are designed to measure math-gender stereotypes with binary (yes/no) responses. We convert these questions into three binary variables: self math-gender stereotype, perceived parental math-gender stereotype, and perceived societal math-gender stereotype. If the response indicated that boys are not better at math than girls, the variable is set to 0. Conversely, if the response indicated that boys are better at math, the variable is set to 1. 

As data on math-gender stereotypes is available only in the first wave, and a fixed effects model requires data from both waves to capture changes over time, we introduced a novel independent variable. For each student, this variable is initialized to 0 in the first wave, and then, in the second wave, it takes on the value of the student’s math-gender stereotype from the first wave. This approach allows us to examine whether math-gender stereotypes while accounting for first-wave math scores in a fixed-effects model, have an additional impact on second-wave math scores.

In the full sample, 53% of students believe boys are better at math than girls, 42.9% believe their parents hold this view, and 55.1% believe most people around them share this belief. Among female students, these proportions are 42.5%, 31.4%, and 49.3%, respectively, all below 50%. This indicates that female students do not generally endorse the notion that boys are superior in math, nor do they perceive their parents or peers as subscribing to this stereotype. For male students, these proportions are 62.7%, 53.5%, and 60.5%, respectively, all above 50%. This suggests that a majority of male students still uphold the stereotype that boys excel in math, even though in this sample, female students actually outperform male students in math.

#### 3.2.3. Control Variable

The fixed-effects model accounts for the influence of time-invariant factors, so the control variables in this study include factors that vary over time. These factors include individual-level variables such as health status, confidence about the future, ease of current math learning, cognitive abilities, and family-level variables such as economic conditions and parental educational expectations.

#### 3.2.4. Instrumental Variable

To address potential endogeneity issues, this study enhances the fixed-effects model by incorporating instrumental variables. The selected instrumental variable is the difference in math performance between male and female students in the first wave within the same classroom. This variable is constructed by calculating the proportion of male and female students who rank in the top 50% of their classroom’s math performance during the first wave.

Following a similar approach to that used for the independent variables, the instrumental variable is defined as follows: it is initially set to 0 for each student in the first wave, and in the second wave, it reflects the value of the within-class difference between male and female first-wave math performance. 

### 3.3. Model Specification

#### 3.3.1. Fixed-Effects Model

This study utilizes data from two waves, 2013–2014 and 2014–2015, to construct a fixed-effects model at the student level. The dependent variable is the math score, and the independent variable is the math-gender stereotype. Other observable factors that could influence math scores are controlled for as covariates in the model, while unobservable factors are accounted for in the error term. Since these unobservable factors may simultaneously affect both the math-gender stereotype and students’ math scores, estimation bias might arise. The fixed-effects model helps mitigate endogeneity issues stemming from non-time-varying factors correlated with the explanatory variable. 

The model is specified as follows:

(1) $${stdmat}_{i}=\alpha_{0}+\alpha_{1}{stereotype}_{i}+{Χ}_{i}\alpha_{2}+\epsilon_{i}$$

The dependent variable, denoted as ${stdmat}_{i}$, represents the standardized math score of student i at the school level. The independent variable, ${stereotype}_{i}$, represents the student’s math-gender stereotype. In the first wave, the value is 0, and in the second wave, it is a dummy variable: 0 if the student disagrees with the notion that males are better at math, and 1 if they agree. ${Χ}_{i}$ represents all control variables. $\epsilon_{i}$ stands for the student-level random error term. The coefficient $\alpha_{1}$ reflects the impact of stereotypes on students’ math scores.

#### 3.3.2. Integration of Fixed-Effects Model with Instrumental Variable

To address potential endogeneity from time-varying unobservable factors, this study enhances the fixed-effects model by introducing an instrumental variable: the first-wave difference between male and female math scores within the same class. This instrumental variable is relevant because it is significantly correlated with the math-gender stereotype. Intuitively, if male students in a class generally outperform their female counterparts or a higher proportion of males achieve excellent scores, both male and female students are more likely to believe that males are better at math. Statistically, when regressing the math-gender stereotype on the difference in male and female math scores while controlling for other variables, the instrument shows significance for both genders and all three stereotype categories (see Table 2).

Moreover, this variable does not directly impact individual students’ math scores, satisfying the exclusion restriction condition. The within-class difference in math scores pertains to the overall performance of male and female groups rather than specific individuals, making it unlikely to directly influence a single student’s math score.

The two-stage least squares (2SLS) regression model under the fixed-effects framework is specified as follows:

(2) $${stereotype}_{ic}=\beta_{0}+\beta_{1}{gap}_{ic}+{Χ}_{ic}\beta_{2}+\epsilon_{ic}$$

(3) $${stdmat}_{ic}=\gamma_{0}+\gamma_{1}\hat{{stereotype}_{ic}}+{Χ}_{ic}\gamma_{2}+\epsilon_{ic}$$

Equation (2) represents the first-stage regression while Equation (3) represents the second-stage regression.

In Equation (2), the dependent variable is the math stereotype, regressed on the instrumental variable (${gap}_{ic}$), which represents the first-wave difference in male and female math scores within the same class. In Equation (3), the dependent variable is the standardized math score, regressed on the predicted values of stereotypes from the first stage. ${Χ}_{ic}$ represents all control variables. $\epsilon_{ic}$ stands for the student-level random error term. The coefficient $\gamma_{1}$ reflects the impact of stereotypes on students’ math scores.

## 4. Results

Given the contrasting nature of math-gender stereotypes—where boys are perceived as proficient in math and girls are perceived as less proficient—the impact of these stereotypes on math scores should be analyzed separately for each gender. Therefore, this study applies the models described earlier to investigate male and female students individually. Additionally, we consider that the impact of math-gender stereotypes might differ among students with varying academic performance. For each gender, we initially analyzed the overall influence of stereotypes on all students. Subsequently, we divide students based on their first-wave math scores into those in the upper half and those in the lower half. This division allows us to analyze the effects of stereotypes on both subgroups separately. Furthermore, math-gender stereotypes are categorized into three types. For each category of independent variables, we use both the fixed-effects model and a combined analysis of the fixed-effects model with instrumental variables.

### 4.1. Effect of Math-Gender Stereotype on Female Students’ Performance

To understand the impact of math-gender stereotypes on female students’ math scores, we first examine this influence using the entire female sample. We then further divide the female students into two groups based on their first-wave math scores to analyze whether the effect of math-gender stereotypes differs among these subgroups.

#### 4.1.1. Effect of Math-Gender Stereotype on All Female Students

Table 3 illustrates the regression of standardized math scores on three categories of math-gender stereotypes: self-perceived, parental, and societal. The first two columns present self-perceived math-gender stereotypes, the middle two columns show parental math-gender stereotypes, and the last two columns display societal math-gender stereotypes. We use two models: odd-numbered columns show fixed-effects models, while even-numbered columns show fixed-effects models combined with instrumental variables. We control for additional independent variables, such as cognitive abilities and parental educational expectations, with standard errors clustered at the class level.

The results across all six columns in Table 3 indicate a consistently negative impact of math-gender stereotypes on female students’ math scores. This is true for both the fixed-effects model and the fixed-effects model with instrumental variables, regardless of the stereotype type. Although the fixed-effects models alone are not statistically significant (columns 1, 3, and 5), incorporating instrumental variables reveals a statistically significant negative impact (columns 2, 4, and 6). This suggests that using instrumental variables mitigates endogeneity concerns for the entire group of female students, showing that math-gender stereotypes negatively affect their math scores. Specifically, female students who endorse the stereotype that boys are better at math than girls tend to have lower math scores, consistent across all three types of stereotypes.

Regarding the magnitude of the effects of the three types of stereotypes, societal math-gender stereotypes have the most pronounced impact on female students. Those who believe that people around them think boys are better at math than girls experience a decrease of 0.345 in standardized math scores compared to those who do not hold this belief. Following this, self-perceived math-gender stereotypes have the next most significant impact. Female students who believe that boys are better at math than girls encounter a reduction of 0.272 in standardized math scores compared to those who do not share this belief. Lastly, parental math-gender stereotypes show the smallest impact. Female students who believe that their parents think boys are better at math than girls experience a decrease of 0.245 in standardized math scores compared to those who do not hold this belief. This ranking aligns with the prevalence of these stereotypes among female students, with agreement proportions for these stereotypes being 49.3%, 42.5%, and 31.4%, respectively.

#### 4.1.2. Effect of Math-Gender Stereotype on Top-Performing Female Students

In Table 4, we examine the impact of math-gender stereotypes on female students with higher math performance. The results show that the coefficients in all six columns are negative, indicating a negative influence of math-gender stereotypes on high-performing female students. However, none of these coefficients are statistically significant. This suggests that math-gender stereotypes do not have a significant impact on the math scores of female students who perform well in math.

#### 4.1.3. Effect of Math-Gender Stereotype on Lower-Performing Female Students

We extended our analysis to female students whose first-wave math scores were in the bottom half in Table 5. The findings are consistent with the results for the overall sample of female students. The coefficients in all six columns are negative. While the fixed-effects models alone are not statistically significant, the inclusion of instrumental variables makes them significant. This implies that, after addressing endogeneity concerns, all three types of math-gender stereotypes negatively impact the math scores of lower-performing female students.

Furthermore, the relative influence of the three types of math-gender stereotypes on the math scores of lower-performing female students mirrors the results for all female students. Specifically, societal math-gender stereotypes have the most pronounced negative effect, followed by self-perceived math-gender stereotypes, and lastly, parental math-gender stereotypes.

Since the results for lower-performing female students align with those of the overall female student group, and there are no significant effects of math-gender stereotypes among higher-performing female students, we can conclude that the overall results for female students are primarily driven by the lower-performing group.

#### 4.1.4. Summary of Results for Female Students

For female students, we have two main findings: 

Firstly, all three types of math-gender stereotypes significantly and negatively affect the math performance of female students. Specifically, female students who believe that boys are better at math than girls tend to achieve lower math scores compared to those who do not hold this belief. Moreover, this negative impact is particularly noticeable among female students in the lower-performing group.

Secondly, through a detailed comparison of coefficient magnitudes, we have established a clear hierarchy regarding the influence of the three types of math-gender stereotypes on female students’ math performance. This hierarchy corresponds to the prevalence of these stereotypes among female students. Specifically, the impact hierarchy is as follows: societal math-gender stereotype > self math-gender stereotype > parental math-gender stereotype. Importantly, this order mirrors the severity ranking of these stereotypes among female students—the prevalence rates of agreement with these stereotypes among female students also follow this sequence: societal math-gender stereotype > self math-gender stereotype > parental math-gender stereotype. This consistency aligns intuitively: the more prevalent a stereotype is, the greater its detrimental effect on female math performance.

### 4.2. Effect of Math-Gender Stereotype on Male Students’ Performance

Math-gender stereotypes, which suggest that boys are better at math, may also influence the performance of male students. Following a methodology similar to that used for female students, we initially analyzed the entire male student population. Subsequently, we divide the male students based on their first-wave math scores into two groups: the top-performing half and the slightly weaker-performing half, conducting separate analyses for each subgroup. We examine the impact of the three types of stereotypes using both fixed effects models and fixed effects models combined with instrumental variables.

#### 4.2.1. Effect of Math-Gender Stereotype on All Male Students

Initially, when considering all male students, the coefficients in Table 6 are positive, but they are not statistically significant. This suggests that none of the three types of stereotypes significantly impact the male student population.

#### 4.2.2. Effect of Math-Gender Stereotype on Top-Performing Male Students

Next, we analyze male students who scored in the top half. The results in Table 7 show that across all three types of stereotypes, male students who believe that boys are better at math than girls tend to achieve higher math scores compared to those who do not hold this belief. This finding holds true for both the fixed effects model and the fixed effects model with instrumental variables.

When comparing the coefficients that represent the impacts of the three types of stereotypes, we observe a consistent pattern similar to the results for female students. Among male students, the societal math-gender stereotype has the strongest impact: male students who perceive that most people around them believe boys are superior in math score 0.772 points higher on the standardized math test (see column (6)). The next most influential is their own math-gender stereotype, with a coefficient of 0.754 (see column (2)), while the parental math-gender stereotype has the least influence, with a coefficient of 0.491.

#### 4.2.3. Effect of Math-Gender Stereotype on Lower-Performing Male Students

We conducted an analysis on male students who scored in the lower half of math scores in the first wave. The results in Table 8 show that for male students with lower scores, agreeing with the stereotype that boys are better at math than girls does not lead to any significant improvement in their math scores compared to those who do not agree with this stereotype.

#### 4.2.4. Summary of Results for Male Students

In summary, the impact of math-gender stereotypes on male students’ math scores reveals a nuanced pattern. On one hand, these stereotypes only demonstrate a positive effect on male students with relatively higher math scores, whereas there is no significant impact observed among male students with slightly weaker scores or across the entire male student population. On the other hand, among male students with relatively higher math scores, comparing the magnitudes of coefficients reveals a hierarchy in the influence of the three types of math-gender stereotypes on their math scores: societal math-gender stereotype > individual math-gender stereotype > parental math-gender stereotype. This ranking aligns with the observed order of influence among female students.

### 4.3. Robustness Check

The math-gender stereotype implies that boys are better at math than girls. Therefore, the expectation is that this stereotype should only affect students’ math performance and not their Chinese performance. To validate the findings related to the impact of math-gender stereotypes on math performance, we utilize standardized Chinese scores as an alternative dependent variable, serving as a placebo test. The results from Table 9 show that, regardless of gender or sample type (full sample or sub-samples), none of the three types of math-gender stereotypes yield significant effects on Chinese scores. Consequently, the conclusion regarding the influence of math-gender stereotypes on students’ math performance remains robust.

Some may also wonder if dividing the male and female groups into high and low performers using a set math cut score would yield different results than our original method. To address this concern, we add a robustness check. Specifically, we first calculate the median of standardized math scores for all students in our sample. Students with scores above the median are classified as high performers, while those with scores below the median are classified as low performers. We then analyze the data separately for high performers and low performers within each gender. The results of this analysis are consistent with our main findings. As shown in Table 10, the effect of stereotypes was particularly detrimental to low-performing females while benefiting high-performing males. This robustness check confirms that our primary results are stable and not sensitive to the method of dividing the subgroups.

## 5. Discussion

Consistent with the conclusions of most previous studies, this research finds that math-gender stereotypes lower math performance among female students and enhance performance among male students in a representative sample of seventh-grade students (average age 13) in China. Interestingly, the impact on girls is observed only among low achievers, while the impact on boys is seen only among high achievers. It is worth exploring why no significant impact was found among high-achieving girls. Some studies suggest that female participants with a higher level of domain identification (i.e., a greater interest in mathematics) are more severely affected by stereotype threat (Hyde and Mertz 2009; Steele 1997). Additionally, experiments conducted on high-achieving girls have shown the negative impacts of stereotypes (Schmader and Johns 2003; Schmader et al. 2008). However, Flore et al. (2018) did not find the effect of stereotype threat among high-achieving girls in the Netherlands (Nollenberger et al. 2016). They proposed potential explanations such as cross-cultural differences (i.e., gender stereotypes have less impact on test performance in Dutch society), generational differences (i.e., the current generation of students is less sensitive to stereotype threat), or other unknown theoretical explanations. We believe that their proposed cross-cultural explanation might also account for our findings, suggesting that in the specific cultural context of China, high-achieving girls are less affected by stereotypes, warranting further investigation.

Moreover, the finding that stereotypes enhance the performance of high-achieving boys supports Ellis’s “Greater Male Variability Hypothesis”, which posits that males exhibit higher variability in physiological and psychological traits, including intelligence (Ellis 1911). This implies that males are more prominently represented at both the highest and lowest ends of trait distributions. Thus, stereotypes increasing the performance of high-achieving boys could further widen the variance in boys’ math performance.

Among the three types of stereotypes examined, perceived societal stereotypes have the most significant impact. Previous research has found a correlation between perceived societal stereotypes and self-stereotypes, as social knowledge is transmitted from influential adults to children, who then use their perceptions of adults’ beliefs to form views about their social group (Kurtz-Costes et al. 2014). While the literature does not typically compare the relative impacts of societal and self-stereotypes on math performance, our findings indicate that societal stereotypes have a greater impact on both boys and girls. The literature also confirms the negative impact of societal gender stereotypes on girls’ math performance (Justicia-Galiano et al. 2023). Therefore, efforts to eliminate stereotypes should focus not only on self-stereotypes but also on societal stereotypes.

Regarding ways to mitigate stereotypes, existing research has primarily explored how to reduce self-stereotypes among women, including both laboratory and school-based interventions. Laboratory methods include training women to adopt a more positive attitude toward math (Forbes and Schmader 2010), conducting verbal tests before math exams (Smeding et al. 2013), informing girls that math ability can be developed (Schmader and Johns 2003), suppressing stereotype-related thoughts (Logel et al. 2009), and introducing female role models (Shin et al. 2016; Leroy et al. 2022). In China, Zhao et al. (2018) conducted a three-month intervention program in classrooms aimed at changing adolescent girls’ collective representations, situational cues, and personal characteristics, which ultimately reduced gender stereotypes (Zhao et al. 2018). Future research should continue to explore effective intervention strategies in real-world settings such as classrooms or schools to reduce stereotypes. Additionally, addressing societal gender stereotypes remains a long-term challenge worth investigating.

This study has several limitations and suggests directions for future research:

First, this study uses data from the China Education Panel Survey (CEPS) collected between 2013 and 2015. These data are somewhat dated, and social and educational environments may have changed over the past decade. However, since the data cover students from various regions and backgrounds, providing high representativeness and quality, we believe they still reveal fundamental patterns and trends in the impact of stereotypes on math performance. Future studies could use newly available data to validate and supplement our findings, enhancing the understanding of this issue. However, it is also necessary to investigate the impact of stereotypes using updated data within the Chinese context.

Second, while the data’s representativeness is a significant advantage, it lacks the causal inference strength of experimental data. We have employed fixed effects models and instrumental variable models to reduce endogeneity, but the results should still be interpreted with caution. Future research should incorporate experimental data to study the impact of stereotypes on math performance more robustly.

Third, the measure of stereotypes used in this study relies on direct questions, which can be leading. Although studies on math-gender stereotypes typically use direct questions, few have included indirect questions (Martinot et al. 2012; Nowicki and Lopata 2017). Direct questions can be influenced by social desirability bias—the tendency to respond in a manner consistent with cultural norms. Therefore, validating our interpretations is an important target for future research. Additionally, since the question is a binary variable, it may lead to greater error variance. Future studies could consider using a continuous variable to improve measurement accuracy.

Finally, CEPS only collected data on students’ personal-level stereotypes and did not include class-level teacher stereotypes or school-level administrator stereotypes. As a result, we can only examine the impact of individual-level stereotypes and not those at the class or school level. Understanding how stereotypes operate at the class or school level is crucial for a comprehensive analysis of their effects on students’ academic performance. Future research should explore how teacher and administrator beliefs at these broader levels may influence students’ perceptions and performance in mathematics.

## Figures and Tables

**Table 1:** Summary statistics.

	Obs	Mean	Sd	Min	Max
Panel A: full sample					
Math score in the first wave	2007	75.274	27.985	3	149
Math score in the second wave	1926	75.188	18.939	2	133
Chinese score in the first wave	2006	70.337	30.284	0	145
Chinese score in the second wave	1926	77.270	18.931	0	128
Self math-gender stereotype	1959	0.530	0.499	0	1
Parental math-gender stereotype	1955	0.429	0.495	0	1
Societal math-gender stereotype	1946	0.551	0.497	0	1
Panel B: female sample					
Math score in the first wave	957	77.446	27.133	6	149
Math score in the second wave	917	73.540	28.641	0	130
Chinese score in the first wave	958	79.308	17.194	6	149
Chinese score in the second wave	917	81.502	16.939	4	128
Self math-gender stereotype	937	0.425	0.494	0	1
Parental math-gender stereotype	933	0.314	0.464	0	1
Societal math-gender stereotype	929	0.493	0.500	0	1
Panel C: male sample					
Math score in the first wave	1050	73.293	28.609	3	146
Math score in the second wave	1048	71.423	19.670	2	133
Chinese score in the first wave	1009	67.426	31.435	0	145
Chinese score in the second wave	1009	73.425	19.815	0	124
Self math-gender stereotype	1022	0.627	0.483	0	1
Parental math-gender stereotype	1022	0.535	0.499	0	1
Societal math-gender stereotype	1017	0.605	0.488	0	1

**Table 2:** First stage of IV.

Dependent Variable	Self Math-Gender Stereotype	Parental Math-Gender Stereotype	Societal Math-Gender Stereotype
	(1)	(2)	(3)
Panel A: All female students			
Math gender gap within class	0.267 *** (0.019)	0.528 *** (0.034)	0.203 *** (0.019)
Other controls	Y	Y	Y
Fixed effects	Y	Y	Y
N	1195	1195	1195
Panel B: All male students			
Math gender gap within class	0.398 *** (0.030)	0.192 *** (0.015)	0.141 *** (0.015)
Other controls	Y	Y	Y
Fixed effects	Y	Y	Y
N	1286	1286	1286

* notes: * *p* < 0.10; ** *p* < 0.05; *** *p* < 0.01. Robust standard errors are reported in parentheses, and control variables are included. Results are presented for all female students in panel A and for all male students in panel B.

**Table 3:** Effect of math-gender stereotype on all female students.

Specification	FE	FE + IV	FE	FE +IV	FE	FE + IV
Sample	All female students					
Dependent variable	Standardized math scores					
	(1)	(2)	(3)	(4)	(5)	(6)
Self math-gender stereotype	−0.020 (0.040)	−0.272 ** (0.101)				
Parental math-gender stereotype			−0.011 (0.041)	−0.245 ** (0.085)		
Societal math-gender stereotype					−0.077 (0.042)	−0.345 ** (0.131)
Other controls	Y	Y	Y	Y	Y	Y
Fixed effects	Y	Y	Y	Y	Y	Y
N	1195	1195	1195	1195	1195	1195
R^2^	0.016	0.013	0.012	0.009	0.011	0.010

Notes: A fixed-effect model and a fixed-effect model combined with an instrumental variable are used. Robust standard errors are reported in parentheses, and control variables are included. * *p* < 0.10; ** *p* < 0.05; *** *p* < 0.01. Results are presented for all female students.

**Table 4:** Effect of math-gender stereotype on top-performing female students.

Specification	FE	FE + IV	FE	FE + IV	FE	FE + IV
Sample	Top half female students					
Dependent variable	Standardized math scores					
	(1)	(2)	(3)	(4)	(5)	(6)
Self math-gender stereotype	−0.090 (0.048)	−0.078 (0.120)				
Parental math-gender stereotype			−0.069 (0.051)	−0.047 (0.111)		
Societal math-gender stereotype					−0.063 (0.050)	−0.101 (0.156)
Other controls	Y	Y	Y	Y	Y	Y
Fixed effects	Y	Y	Y	Y	Y	Y
N	598	598	598	598	598	598
R^2^	0.055	0.054	0.048	0.047	0.070	0.067

Notes: A fixed-effect model and a fixed-effect model combined with an instrumental variable are used. Robust standard errors are reported in parentheses, and control variables are included. * *p* < 0.10; ** *p* < 0.05; *** *p* < 0.01. Results are presented for the top half of female students.

**Table 5:** Effect of math-gender stereotype on lower-performing female students.

Specification	FE	FE + IV	FE	FE + IV	FE	FE + IV
Sample	Bottom half female students					
Dependent variable	Standardized math scores					
	(1)	(2)	(3)	(4)	(5)	(6)
Self math-gender stereotype	−0.100 * (0.059)	−0.637 ** (0.156)				
Parental math-gender stereotype			−0.095 * (0.058)	−0.497 ** (0.116)		
Societal math-gender stereotype					−0.053 (0.064)	−0.780 *** (0.202)
Other controls	Y	Y	Y	Y	Y	Y
Fixed effects	Y	Y	Y	Y	Y	Y
N	597	597	597	597	597	597
R^2^	0.097	0.091	0.097	0.087	0.092	0.085

Notes: A fixed-effect model and a fixed-effect model combined with an instrumental variable are used. Robust standard errors are reported in parentheses, and control variables are included. * *p* < 0.10; ** *p* < 0.05; *** *p* < 0.01. Results are presented for the bottom half of female students.

**Table 6:** Effect of math-gender stereotype on all male students.

Specification	FE	FE + IV	FE	FE + IV	FE	FE + IV
Sample	All male students					
Dependent variable	Standardized math scores					
	(1)	(2)	(3)	(4)	(5)	(6)
Self math-gender stereotype	0.082 * (0.046)	0.205 (0.163)				
Parental math-gender stereotype			0.040 (0.043)	0.154 (0.122)		
Societal math-gender stereotype					0.072 (0.046)	0.209 (0.161)
Other controls	Y	Y	Y	Y	Y	Y
Fixed effects	Y	Y	Y	Y	Y	Y
N	1286	1286	1286	1286	1286	1286
R^2^	0.012	0.003	0.009	0.0002	0.011	0.004

Notes: A fixed-effect model and a fixed-effect model combined with the instrumental variable are used. Robust standard errors are reported in parentheses, and control variables are included. * *p* < 0.10; ** *p* < 0.05; *** *p* < 0.01. Results are presented for all male students.

**Table 7:** Effect of math-gender stereotype on top-performing male students.

Specification	FE	FE + IV	FE	FE + IV	FE	FE + IV
Sample	Top half male students					
Dependent variable	Standardized math scores					
	(1)	(2)	(3)	(4)	(5)	(6)
Self math-gender stereotype	0.277 *** (0.056)	0.754 ** (0.241)				
Parental math-gender stereotype			0.154 ** (0.052)	0.491 ** (0.154)		
Societal math-gender stereotype					0.219 *** (0.056)	0.772 ** (0.253)
Other controls	Y	Y	Y	Y	Y	Y
Fixed effects	Y	Y	Y	Y	Y	Y
N	643	643	643	643	643	643
R^2^	0.064	0.061	0.059	0.056	0.076	0.069

Notes: A fixed-effect model and a fixed-effect model combined with an instrumental variable are used. Robust standard errors are reported in parentheses, and control variables are included. * *p* < 0.10; ** *p* < 0.05; *** *p* < 0.01. Results are presented for the top half of male students.

**Table 8:** Effect of math-gender stereotype on lower-performing male students.

Specification	FE	FE + IV	FE	FE + IV	FE	FE + IV
Sample	Bottom half male students					
Dependent variable	Standardized math scores					
	(1)	(2)	(3)	(4)	(5)	(6)
Self math-gender stereotype	0.076 (0.072)	0.192 (0.231)				
Parental math-gender stereotype			0.045 (0.071)	0.159 (0.192)		
Societal math-gender stereotype					0.053 (0.072)	0.189 (0.223)
Other controls	Y	Y	Y	Y	Y	Y
Fixed effects	Y	Y	Y	Y	Y	Y
N	643	643	643	643	643	643
R^2^	0.062	0.055	0.060	0.053	0.064	0.054

Notes: A fixed-effect model and a fixed-effect model combined with an instrumental variable are used. Robust standard errors are reported in parentheses, and control variables are included. * *p* < 0.10; ** *p* < 0.05; *** *p* < 0.01. Results are presented for the bottom half of male students.

**Table 9:** Placebo test.

Specification	FE	FE + IV	FE	FE + IV	FE	FE + IV
Dependent variable	Standardized Chinese scores					
	(1)	(2)	(3)	(4)	(5)	(6)
Panel A						
Sample	All female		Top half female		Bottom half female	
Self math-gender stereotype	−0.006 (0.043)	0.012 (0.124)	−0.014 (0.056)	−0.025 (0.173)	0.038 (0.062)	0.025 (0.170)
Parental math-gender stereotype	0.015 (0.040)	0.004 (0.098)	−0.012 (0.053)	−0.015 (0.133)	0.050 (0.058)	0.008 (0.138)
Societal math-gender stereotype	−0.022 (0.041)	0.006 (0.084)	−0.049 (0.057)	−0.031 (0.123)	0.005 (0.058)	0.017 (0.108)
Panel B						
Sample	All male		Top half male		Bottom half male	
Self math-gender stereotype	0.038 (0.051)	0.057 (0.180)	−0.001 (0.061)	−0.109 (0.248)	0.035 (0.083)	0.111 (0.258)
Parental math-gender stereotype	−0.013 (0.052)	0.024 (0.184)	−0.042 (0.063)	−0.183 (0.249)	−0.013 (0.082)	0.111 (0.267)
Societal math-gender stereotype	−0.036 (0.049)	0.017 (0.137)	−0.147 (0.057)	−0.120 (0.160)	0.078 (0.081)	0.092 (0.220)

Notes: A fixed-effect model and a fixed-effect model combined with an instrumental variable are used. Robust standard errors are reported in parentheses, and control variables are included. * *p* < 0.10; ** *p* < 0.05; *** *p* < 0.01.

**Table 10:** Robustness check.

Specification	FE	FE + IV	FE	FE + IV	FE	FE + IV	FE	FE + IV
Dependent variable	Standardized math scores							
	(1)	(2)	(3)	(4)	(5)	(6)	(7)	(8)
Sample	Above-median female		Below-median female		Above-median male		Below-median male	
Self math-gender stereotype	−0.074 (0.062)	−0.094 (0.135)	−0.060 (0.079)	−0.497 * (0.186)	0.225 ** (0.098)	0.731 ** (0.276)	0.068 (0.077)	0.178 (0.225)
Parental math-gender stereotype	−0.056 (0.053)	−0.037 (0.106)	−0.085 (0.078)	−0.344 * (0.197)	0.141 ** (0.062)	0.527 ** (0.209)	0.031 (0.082)	0.137 (0.178)
Societal math-gender stereotype	−0.067 (0.058)	−0.083 (0.136)	−0.107 (0.093)	−0.768 ** (0.293)	0.198 ** (0.086)	0.755 ** (0.277)	0.059 (0.088)	0.172 (0.201)

Notes: A fixed-effect model and a fixed-effect model combined with an instrumental variable are used. Robust standard errors are reported in parentheses, and control variables are included. * *p* < 0.10; ** *p* < 0.05; *** *p* < 0.01.

## Data Availability

The original data presented in the study are openly available in http://ceps.ruc.edu.cn.
